# Methamphetamine-induced cardiotoxicity: in search of protective transcriptional mechanisms

**DOI:** 10.1007/s00059-024-05279-6

**Published:** 2024-10-25

**Authors:** Kristin Annawald, Katrin Streckfuss-Bömeke, Thomas Meyer

**Affiliations:** 1 Department of Psychosomatic Medicine and Psychotherapy, University of Göttingen, Waldweg 33, 37073 Göttingen, Germany; 2 Institute of Pharmacology and Toxicology, University of Würzburg, Würzburg, Germany

**Keywords:** Methamphetamine hydrochloride, Signal transduction, STAT transcription factors, Dilated cardiomyopathy, Cardioprotective agents, Metamphetaminhydrochlorid, Signaltransduktion, STAT-Transkriptionsfaktoren, Dilatative Kardiomyopathie, Kardioprotektive Substanzen

## Abstract

Crystalline methamphetamine hydrochloride is an illegal drug with a high addictive potential, better known by its colloquial name “ice” or “crystal meth”. The abuse of this drug has led to significant health problems worldwide. Like other amphetamine-type stimulants, chronic consumption of methamphetamine leads to direct toxic effects on the central nervous system, causing cognitive impairment, depressive behavior, and other severe neurological or psychiatric symptoms. Besides its neurotoxicity, the drug exhibits numerous deleterious effects on the cardiovascular system, including hypertension, accelerated atherosclerosis, vasospasm-induced acute coronary syndromes, sudden cardiac death, and dilated cardiomyopathy with congestive heart failure and left ventricular dysfunction. The excessive release of catecholamines upon methamphetamine exposure causes vasoconstriction and vasospasm, which ultimately lead to hypertension, tachycardia, endothelial dysfunction, and cardiotoxicity. While numerous studies have focused on transcription factors expressed in the brain that cause the neurotoxic effects of the drug, much less is known about transcription factors involved in the development of methamphetamine-induced heart failure. In this article, we provide an overview of the Janus kinase–signal transducer and activator of transcription 3 (JAK–STAT3) pathway involved in ischemia/reperfusion injury in the myocardium, which may be activated by the vasospasm-inducing action of the drug. However, much more work is needed to decipher the precise role of STAT protein family members, including the potentially cardioprotective STAT3, in the pathogenesis of methamphetamine-induced cardiotoxicity.

## Abstract (de)

Kristallines Methamphetaminhydrochlorid ist eine illegale, unter ihrem umgangssprachlichen Namen „Ice“ oder „Crystal Meth“ besser bekannte Droge mit hohem Suchtpotenzial. Der Missbrauch dieser Droge hat weltweit zu erheblichen gesundheitlichen Problemen geführt. Wie andere amphetaminartige Stimulanzien führt die chronische Aufnahme von Methamphetamin zu direkten toxischen Wirkungen auf das zentrale Nervensystem und verursacht kognitive Beeinträchtigungen, depressives Verhalten und andere schwere neurologische oder psychiatrische Symptome. Neben der Neurotoxizität hat die Droge zahlreiche schädliche Auswirkungen auf das Herz-Kreislauf-System, darunter Bluthochdruck, beschleunigte Atherosklerose, durch Vasospasmen ausgelöste akute Koronarsyndrome, plötzlicher Herztod und dilatative Kardiomyopathie mit kongestiver Herzinsuffizienz und linksventrikulärer Dysfunktion. Die übermäßige Freisetzung von Katecholaminen nach Methamphetaminexposition verursacht Vasokonstriktion und Vasospasmen, die letztlich zu Hypertonus, Tachykardie, endothelialer Dysfunktion und Kardiotoxizität führen. Während zahlreiche Studien zu Transkriptionsfaktoren existieren, die im Gehirn exprimiert werden und die neurotoxischen Wirkungen der Droge vermitteln, ist weitaus weniger über solche Transkriptionsfaktoren bekannt, die an der Entwicklung einer durch Methamphetamin induzierten Herzinsuffizienz beteiligt sind. Im vorliegenden Artikel wird ein Überblick über den JAK-STAT3-Signalweg gegeben, der an Ischämiereperfusionsereignissen beteiligt ist und protektiv auf die vasospasmusauslösende Reaktion der Droge wirkt. Jedoch sind noch viele weitere Studien erforderlich, um die genaue Rolle der Mitglieder der STAT-Proteinfamilie, insbesondere des potenziell kardioprotektiven STAT3, bei der Pathogenese der durch Methamphetamin induzierten Kardiotoxizität zu entschlüsseln.

## Toxicology of amphetamine and methamphetamine

Many countries are currently experiencing an opioid epidemic, which was triggered in the late 1990s by an unforeseen increase in opioids being prescribed for non-therapeutic purposes [1]. The abuse of psychoactive drugs, such as amphetamine, also known as α‑methylphenethylamine, methamphetamine (*N*-methyl-α-methylphenethylamine), 3,4-methylenedioxy-methamphetamine (MDMA), and other related substances, has also increased in recent decades, leading to significant additional public health problems [2]. Addiction to psychostimulants is usually the result of recreational use and is associated with both cardiovascular complications and psychiatric symptoms, including methamphetamine-induced psychosis with paranoia and hallucinations as well as anxiety and/or depression. The use of amphetamine-type stimulants is linked to a higher risk for violent behavior and increased suicidality [3]. Medical indications for the administration of amphetamine-type stimulants have also been described, which include attention-deficit/hyperactivity disorder (ADHD), narcolepsy, and severe obesity.

**Fig. 1:**
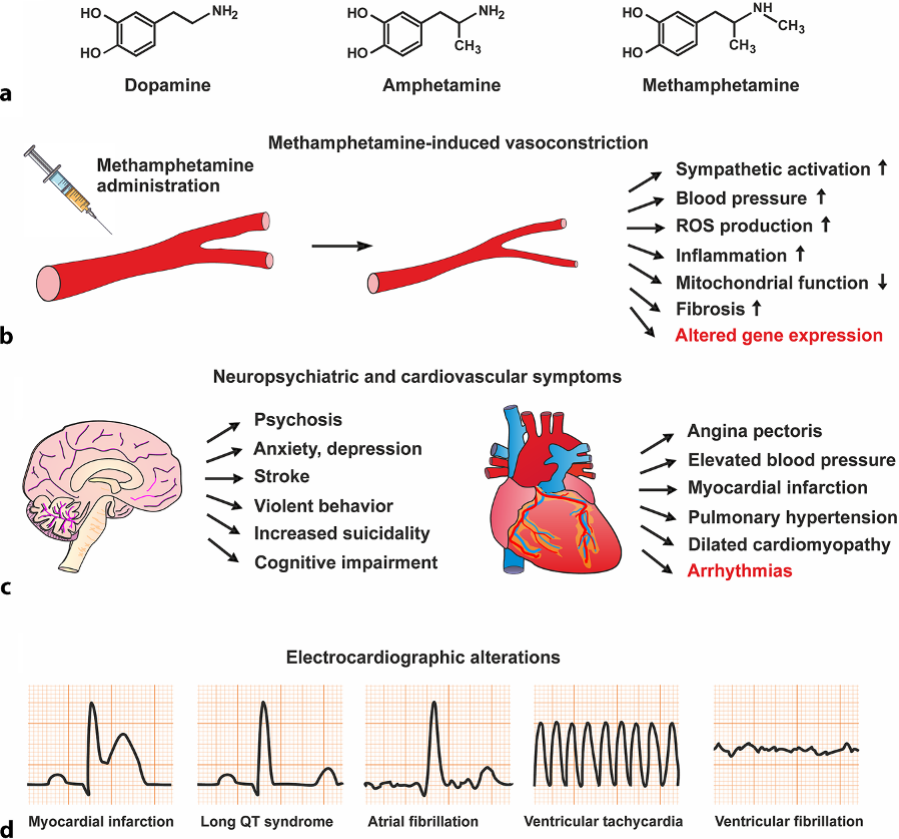
**a** Chemical structures of the endogenous neurotransmitter dopamine and the two related psychostimulants methamphetamine and amphetamine. **b** Physiological effects of methamphetamine-induced vasoconstriction including altered gene expression. **c** List of neuropsychiatric and cardiovascular symptoms associated with the use of amphetamine-type stimulants. **d** Illustration of possible electrocardiographic changes in patients with methamphetamine-induced cardiomyopathy. *ROS* reactive oxygen species

Amphetamine-type stimulants act in multiple organs by promoting catecholamine signaling through a variety of mechanisms. The chemical structure of methamphetamine is similar to the neurotransmitter dopamine and, due to the presence of an additional methyl group in the molecule, it is more lipophilic and has stronger addictive properties than amphetamine (Fig. 1a). Due to the lipophilicity of methamphetamine, the drug can easily cross the blood–brain barrier and the placenta. Routes of administration for crystalline methamphetamine hydrochloride, known colloquially as “ice” or “crystal meth” in the illicit drug market, include oral, intravenous, and intranasal use as well as inhalation when smoking. Administration of methamphetamine or other amphetamine-type stimulants via the nose often results in rhinitis medicamentosa, a drug-induced nasal congestion caused by intermittent vasoconstriction associated with repeated ischemia/reperfusion cycles (Fig. 1b). The chiral, cationic molecule methamphetamine is primarily metabolized by the polymorphic enzyme cytochrome P450 2D6 (CYP2D6) in hepatocytes via *N*-dealkylation, aromatic hydroxylation, and deamination, producing para-hydroxymethamphetamine and amphetamine as intermittent metabolites [4].

## Adverse side effects of amphetamine-type stimulants

Methamphetamine and its main metabolite amphetamine belong to the class of phenethylamines, which stimulate the central nervous system. Amphetamine has been used in the past under the name Benzedrine to treat patients with nasal congestion and major depression. At low doses, amphetamine-type stimulants cause a range of mood effects such as intense euphoria, decreased appetite, increased sexual desire, and increased alertness as well as improved cognitive control with shortened reaction times due to the stimulatory effect on excitatory monoaminergic neurotransmission in the brain.

The amphetamine-type stimulants promote physical endurance, including improved fatigue resistance, tachycardia, and increased muscle strength. The extremely addictive methamphetamine is abused recreationally as a means to improve cognitive and athletic performance, as an aphrodisiac, and/or as a euphoriant. Due to increased arousal and memory consolidation, methamphetamine and its related derivatives have positive effects on task saliency and promote goal-directed behavior, which explain their high potential for abuse among students and young adults to enhance their academic and occupational performance. In summary, the widespread misuse of these psychoactive substances is due to their pharmacological properties that intensify positive emotions and alertness.

At higher concentrations and long-term use, methamphetamine impairs working memory and other cognitive functions, as the psychostimulant causes neurocognitive deficits in various domains of executive functions, indicating the methamphetamine-associated neurotoxicity of this class of drugs ([5]; Fig. 1c). Due to significant psychiatric and somatic comorbidity, amphetamine-type stimulant use is associated with a higher risk of death compared with non-use [6]. Although the mortality rate among users of amphetamine-type stimulants is reported to be higher than in the general population, it is still lower than among opioid users [7]. The increased morbidity and mortality are due to a wide range of adverse effects on the cardiovascular and the nervous system.

Sympathomimetic drugs such as the amphetamine-type stimulants induce severe arterial vasospasms both in the systemic and pulmonary circulation, leading to hypertension, headache, abdominal pain, Raynaud syndrome, finger and toe gangrene, hemorrhagic and ischemic stroke, and arterial dissection, all of which can be considered arterial-related side effects due to vascular spasm (Fig. 1c). Cases of severe arterial vasospasm following methamphetamine administration have been documented both for smaller, peripheral vessels and for large-diameter arteries, including the aorta and coronary arteries [1, 8]. Numerous non-cardiac side effects have been reported, including agitation, decreased seizure threshold, priapism with frequent and prolonged erections, and various gastrointestinal symptoms, blurred vision, and xerostomia.

In addition, amphetamine-type stimulants trigger a variety of cardiac symptoms, including angina pectoris, arrhythmias, myocardial infarction, and decompensated heart failure, which are probably due to recurrent episodes of drug-induced coronary vasospasm ([9]; Fig. 1c). Tissue remodeling in the myocardium is a characteristic feature observed in long-term methamphetamine use, which ultimately leads to severe systolic dysfunction and dilation of the left ventricle. Patients diagnosed with methamphetamine-related cardiomyopathy exhibit an enlarged and dilated heart with reduced contractility and increased left ventricular mass index [10]. Electrocardiographic features associated with methamphetamine-related cardiomyopathy are ST-segment elevation due to myocardial infarction, long QT syndrome, atrial fibrillation, ventricular tachycardia, and rarely ventricular fibrillation (Fig. 1d). Some studies have shown that the reduced left ventricular systolic function improves after prolonged abstinence leading to fewer hospitalizations for heart failure symptoms. This observation suggests that methamphetamine-related cardiomyopathy is reversible in principle [10].

## Biochemical actions of methamphetamine in the heart–brain axis

The increased risk of cardiovascular events reported in crystal meth users is aggravated by unhealthy lifestyle behaviors including tobacco abuse. In addition to its neurotoxicity, chronic use of methamphetamine promotes the formation of atherosclerotic plaques through endothelial activation and cytokine-driven inflammation [1]. On the other hand, methamphetamine has direct toxic effects due to increased oxidative stress, endothelial dysfunction, and altered regional gene expression programs. The drug induces a hyperadrenergic state by inhibiting the catecholamine-degrading enzyme monoamine oxidase, thereby increasing catecholamine levels [1, 5]. By interacting with dopamine (DAT), noradrenaline (NET) and serotonin transporters (SERT) as well as the *N*-methyl-D-aspartate (NMDA) receptor, methamphetamine augments the release of peripheral and central neurotransmitters, such as dopamine, norepinephrine, serotonin (also known as 5‑hydroxytryptamine), and glutamate [5, 11]. Interactions between methamphetamine and the dopamine and norepinephrine transporters have been reported to stimulate the release of catecholamine by modulating receptor activity and preventing the reuptake of extracellularly released catecholamine into cells.

Repeated exposure to amphetamine or its substituted derivatives leads to changes in transcriptional and epigenetic programs in the brain and peripheral organs, including the heart [12]. Important transcription factors expressed in the brain are FBJ murine osteosarcoma viral oncogene homolog B (FosB), nuclear factor-κB (NF-κB), and cAMP response element-binding protein (CREB). In a rat model of chronic methamphetamine administration, persistent upregulation of regional Fos and FosB/∆FosB expression was found in the brain, suggesting a role for the lateral hypothalamus and lateral septum in methamphetamine-seeking behavior [13]. Increased neurotoxicity of methamphetamine was observed in mice deficient in FosB expression [14]. Administration of methamphetamine to rats leads to altered transcriptional responses in the striatum, including recruitment of phosphorylated CREB at *c‑fos, bdnf,* and other promoters [15]. Furthermore, methamphetamine administration resulted in a dose-dependent activation of striatal NF-κB activity, which is attenuated in superoxide dismutase transgenic mice, suggesting a role of reactive oxygen species in the pathogenesis of methamphetamine-induced neurotoxicity [16].

Coelho-Santos and colleagues demonstrated that exposure of cultured N9 microglial cells to methamphetamine induces cell death and decreased cell proliferation in a concentration-dependent manner [17]. The authors showed that interleukin‑6 (IL-6) supplied to microglial cells at low concentrations has a protective effect against drug-induced cell death and that this effect is mediated by the activation of signal transducer and activator of transcription 3 (STAT3). Using a murine model in which the expression of the transcription factor STAT3 was knocked out in astrocytes, Shi and colleagues reported that STAT3 restored the astrocytic capacity of glutamate clearance in the dorsal hippocampal region dCA1 and normalized glutamate levels at dCA1 synapses [18]. This prevented spatial memory impairments following methamphetamine withdrawal.

The widely expressed neuropeptide CART (cocaine- and amphetamine-regulated transcript) has been implicated in stress-induced anxiety-like behavior during abstinence from alcohol and was shown to be upregulated in the striatum following cocaine and amphetamine administration [19, 20]. Using a Langendorff perfusion model, Wang et al. recently showed that stimulation with CART improves cardiac function and reduces myocardial ischemia/reperfusion injury [21]. Pretreatment with CART decreased the ischemia/reperfusion-induced myocardial cell death, downregulated autophagy, and significantly inhibited oxidative stress. The protective effects of CART on myocardial ischemia/reperfusion injury are probably mediated through the phosphoinositide 3‑kinase/protein kinase B (PI3K/AKT) signal pathway, which promotes cell survival and stimulates proliferation in response to extracellular signals and regulates the cell cycle [21].

## The JAK–STAT3 pathway in the heart

Compared with the contribution of brain-expressed transcription factors to addiction and reward processes, much less is known about altered gene expression profiles in methamphetamine-induced cardiotoxicity. Because methamphetamine-induced vasospasm can mimic repetitive ischemia/reperfusion injuries, we hypothesize that STAT3 activation may counteract the adverse effects of methamphetamine-induced cardiotoxicity. In the following, we provide a brief overview of the putative involvement of this cytokine-driven signaling pathway.

**Fig. 2:**
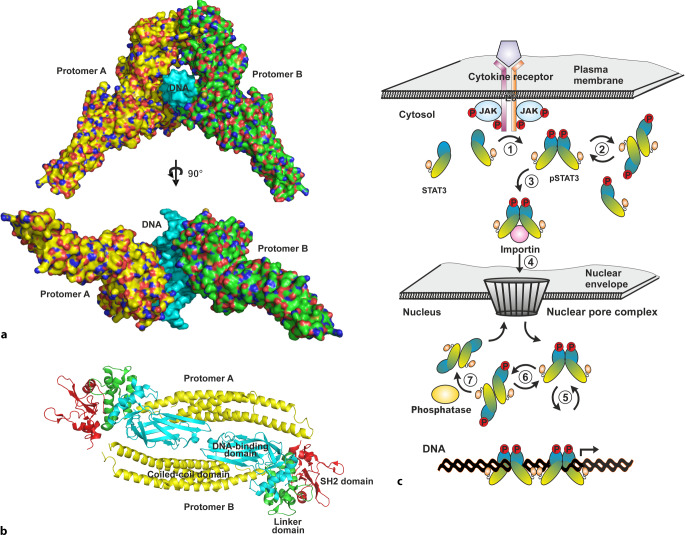
**a** Crystal structure of a parallel signal transducer and activator of transcription 3 (*STAT3*) dimer bound to DNA in orthogonal views. The surface structure is colored according to atom type, with oxygen in red, nitrogen in blue, sulfur in dark yellow, and carbon in either bright yellow or green depending on the protomer. The double-helix structure of DNA is colored in cyan. The crystallographic data were taken from the Protein Data Bank (PDB) file 1BG1 for the STAT3 parallel dimer [27]. **b** Ribbon diagram of an anti-parallel STAT3 dimer. The α‑helical coiled-coil domains are colored in yellow, the DNA-binding domains in cyan, the linker domains in green*,* and the SH2 domains in red. Structural data were from the PDB file 6TLC for STAT3 [29]. Figures **b** and **c** were created with the program PyMOL (DeLano Scientific). **c** Schematic model of the interleukin (IL)-6-induced JAK/STAT3 signaling pathway. Binding of IL‑6 or a related cytokine to the heterodimeric cell surface receptor triggers a series of tyrosine-phosphorylation steps catalyzed by non-covalently bound Janus kinase (*JAK*), including JAK auto-phosphorylation and receptor phosphorylation. The phosphorylated receptor tail recruits STAT3 molecules, which are then phosphorylated at a single tyrosine (*1*). Through spontaneous dissociation and re-association, the activated STAT3 proteins constantly oscillate between a parallel and an antiparallel dimer conformation (*2*). After binding to importins (*3*), phospho-STAT3 dimers are imported into the nucleus via nuclear pore complexes (*4*). In the nucleus, STAT3 proteins modulate gene expression (*5*) and rearrange in an antiparallel dimer conformation (*6*) to be dephosphorylated (*7*)

One important signaling pathway known to alleviate ischemia/reperfusion injury is the Janus kinase (JAK)–STAT3 pathway, which is involved in a variety of different cellular processes, including inflammation, proliferation, cell-cycle regulation, and development, suggesting that it controls cell fate in normal development and under pathophysiological conditions [22]. As the name implies, JAK–STAT3 signal transduction transmits information received from extracellular receptor ligands directly to the transcriptional machinery in the nucleus, without the interaction of small-molecule second messengers. The STAT proteins are a family of evolutionarily conserved transcription factors that were first identified as DNA-binding proteins involved in interferon signaling (Fig. 2a). In humans, seven different members of the STAT family of transcription factors have been identified, namely, STAT1, STAT2, STAT3, STAT4, STAT5a, STAT5b, and STAT6, as well as numerous splice variants of these. The different members of the family are activated by different combinations of cytokines, growth factors, or hormones. Most of the research on STAT proteins in the heart is limited to STAT1 and STAT3, which have antagonistic functions.

All members of the STAT family have a conserved domain structure consisting of a carboxy-terminal transactivating domain and an amino-terminal domain separated from the core domain by a protease-sensitive linker peptide ([22]; Fig. 2b). A distinct hook-shaped domain, formed by the folding of the approximately 130-residue-long amino-terminal domain, promotes the formation of tetramers and their cooperative binding on DNA [23]. The large core domain, which begins at the amino-terminal end with a four-helix bundle involved in protein–protein interactions, consists of multiple structurally different domains [24]. Sequence-specific DNA binding of phosphorylated STAT dimers requires the DNA-binding domain, which has an immunoglobulin fold ([25]; Fig. 2b). The linker region has a distinct α‑helical topology that may be involved in DNA binding [26]. Through reciprocal interactions between the phosphotyrosine of one monomer and the Src homology 2 (SH2) domain of the partner monomer, the SH2 domain promotes binding to the cytokine receptor and the formation of phosphorylated STAT dimers [27, 28]. The major size and sequence differences between the many members of the STAT family are found in the carboxy-terminal transactivating domain, which is removed in many splice variants. Two STAT molecules align either in a parallel dimer conformation capable of binding DNA or in an antiparallel conformation through reciprocal interactions between the coiled-coil domain and the DNA-binding domain ([27, 29]; Fig. 2b).

JAK proteins, named after the mythical Roman god Janus with the two faces, are cytosolic tyrosine kinases that activate STAT proteins by initiating a series of phosphorylation steps (Fig. 2c). Four distinct proteins, known as JAK1, JAK2, JAK3, and TYK2, each with roughly 1200 amino acid residues, are expressed in human cells [30]. Signal transduction by JAK kinases is achieved through the recruitment and subsequent tyrosine phosphorylation of STAT transcription factors. Seven conserved domains, commonly known as JAK homology (JH) domains, are a common property of JAK proteins [30]. As opposed to the neighboring JH2 domain, which seems to be a pseudokinase domain devoid of enzymatic activity, the carboxy-terminal JH1 domain carries complete catalytic activity.

Auto-phosphorylation of non-covalently bound JAK kinases occurs when a ligand binds to the extracellular domain of cytokine receptors and dimerizes the receptor subunits (Fig. 2c). Activated JAKs then phosphorylate tyrosine residues in the cytoplasmic domain of the cytokine receptors, forming docking sites for the SH2 domain of STAT proteins, and subsequently phosphorylate STAT proteins [28, 30]. After the release from the receptor complex, the tyrosine-phosphorylated STATs either homodimerize or heterodimerize before they are translocated to the nucleus in a complex with importin α5, importin β, and RanGTP [31]. In the nucleus, STAT dimers modulate gene transcription by binding to the consensus sequence 5′-TTC(N_4‑6_)GAA-′3, also known as the gamma-activated site (GAS). Finally, specific tyrosine phosphatases dephosphorylate STAT proteins, so that they can leave the nucleus and be rephosphorylated at an active, ligand-bound receptor complex [32]. Furthermore, STATs constantly shuttle back and forth between the cytosolic and nuclear compartments by facilitated diffusion, independent of cytokine stimulation [33].

## Protective role of STAT3 in the heart

STAT3 was initially identified as an oncogene that stimulates cellular transformation and induces a variety of tumors. It was later found that STAT3 activation also plays a role in cardiac remodeling, where the protein is an important modulator of ventricular hypertrophy and regeneration [34, 35]. Myocardial hypertrophy was observed in transgenic mice with cardiac-specific overexpression of STAT3, leading to increased expression of cardiotrophin‑1, α‑myosin heavy chain (MHC), and atrial natriuretic factor (ANF; [36]). Furthermore, when overexpressed in the heart, STAT3 protects mice from doxorubicin-induced cardiomyopathy [36]. The reduced cardiotoxicity of doxorubicin in these mice increased their chance to survive intraperitoneal injections of this cytotoxic anthracycline antibiotic by delaying the onset of heart failure [36]. Notably, STAT3 expression protects against cardiomyocyte injury caused by hypoxia and reoxygenation [37–40]. In cardiac myocytes transfected with a constitutively active STAT3 mutant or stimulated with the STAT3 agonist leukemia inhibitory factor (LIF), increased resistance to hypoxia/reoxygenation-induced cell death was observed. Hilfiker-Kleiner et al. reported that transgenic mice lacking a functional STAT3 gene in the heart exhibited a decrease in myocardial capillary density and an increase in interstitial fibrosis in the first 4 months of life [37]. This was followed by dilated cardiomyopathy, which led to impaired cardiac function and early death. Increased cardiac apoptosis and larger infarct sizes were seen in the STAT3-deficient mice, which were more susceptible to myocardial ischemia/reperfusion damage and infarction [37]. Oshima et al. confirmed that hearts of STAT3-overexpressing mice had more capillaries than those of non-transgenic mice [38].

Ischemic preconditioning led to activation of STAT3 and was associated with a cardioprotective effect, as demonstrated by an improved post-ischemic contractile recovery, a reduced myocardial infarct size, and a lower number of apoptotic cardiomyocytes [37–42]. To mediate protection during ischemic preconditioning, activated STAT3 upregulates the expression of genes that are cardioprotective, such as *Bcl-xl, Vegf* (vascular endothelial growth factor), manganese superoxide dismutase, and metallothionein [38, 42–45]. The STAT3-dependent cytokine IL‑6 plays an obligatory role in late preconditioning by upregulating the expression of the *iNos* (inducible nitric oxide synthase) and *Cox‑2* (cyclooxygenase 2) genes, both of which are under the transcriptional control of STAT proteins [46]. In IL‑6 knockout mice, the ischemia preconditioning-induced tyrosine phosphorylation of STAT3 is significantly inhibited, leading to decreased expression of cyclooxygenase and inducible nitric oxide synthase [46].

In the hearts of mice with cardiac-specific STAT3 deletion, Wegrzyn and colleagues revealed abnormalities in mitochondrial respiration and oxidative phosphorylation [47]. In addition to its function as an inducible cytokine-driven transcription factor in the nucleus, STAT3 regulates the activities of complexes I and II of the electron transport chain in the mitochondria [47, 48]. The finding that mitochondrial STAT3 increases the activity of the electron transport chain is particularly relevant for ischemic postconditioning [43]. It was demonstrated that STAT3 integrates nuclear gene expression and mitochondrial respiration during the self-renewal of pluripotent mouse embryonic stem cells upon stimulation of cells with LIF [49].

## Conclusion

In summary, numerous studies have demonstrated a central role of signal transducer and activator of transcription 3 (STAT3) phosphorylation and gene expression in repeated cycles of ischemia/reperfusion. Similar to the ischemia/reperfusion injury, chronic uptake of methamphetamine is associated with repeated episodes of regional vasospasms followed by consecutive vasodilatation. The functional similarity between methamphetamine-induced cardiotoxicity and ischemia/reperfusion injury suggests that drug-induced vasospasm should also lead to activation of STAT3. Further research is needed to experimentally investigate whether STAT3 activation in methamphetamine-induced cardiotoxicity also has a cardioprotective effect, counteracting the adverse effects of the drug and restoring normal cardiac function after drug discontinuation.

## Abbreviations

CART: cocaine- and amphetamine-regulated transcript
JAK: Janus kinase
LIF: leukemia inhibitory factor
SH2: src homology 2
STAT: signal transducer and activator of transcription

## Declarations

For this article no studies with human participants or animals were performed by any of the authors. All studies mentioned were in accordance with the ethical standards indicated in each case.
